# Effect of seepage conditions on the microstructural evolution of loess across north-west China

**DOI:** 10.1016/j.isci.2022.104691

**Published:** 2022-06-30

**Authors:** Lin Wang, Wen-Chieh Cheng, Wenle Hu, Shaojie Wen, Sen Shang

**Affiliations:** 1 School of Civil Engineering, Xi’an University of Architecture and Technology, Xi’an 710055, China; 2 Shaanxi Key Laboratory of Geotechnical and Underground Space Engineering (XAUAT), Xi’an 710055, China

**Keywords:** Geochemistry, Geology, Mineral-water interface geochemistry

## Abstract

Loess features metastable microstructure and is deemed susceptible to chemical contaminant permeation. However, studies on the loess permeability evolution under water and chemical environments are remarkably limited. In this study, the response of the loess to the water and sodium sulfate seepages was analyzed using the temporal relationship of cations concentration, X-ray diffraction and fluorescence (XRD and XRF), mercury intrusion porosimetry (MIP), and scanning electron microscope (SEM) tests. The permeability evolution characteristics were identified, and its underlying mechanisms were revealed from aspects of the diffuse double layer (DDL) theory and physiochemical actions. The discharge of Mg^2+^ and precipitation of calcium carbonate, referred also to as the dedolomitization, degraded the macro permeability when subjected to the water seepage test. The salt-induced swelling, induced by the intrusion of Na^+^ into the DDL, caused an increase in the micropore fraction under the sodium sulfate seepage test, thereby increasing the macro permeability.

## Highlights

• The *k* evolution of the loess under the water and Na_2_SO_4_ seepages is investigated
• The dedolomitization takes part in the *k* degradation under the water seepage
• The Na^+^ intrusion into the double layer enhances the *k* under the Na_2_SO_4_ seepage

## Graphical abstract

**Figure:**
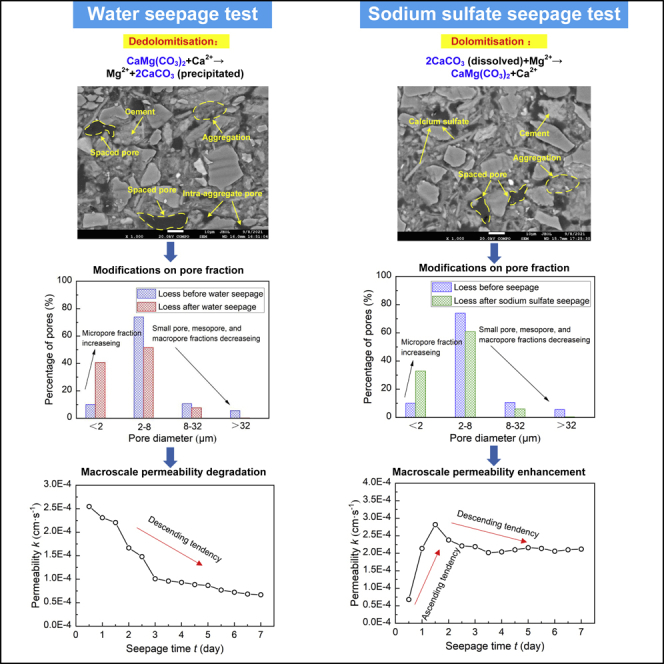

## Introduction

Loess soils are widely distributed in semi-arid and arid regions and are considered susceptible to a sudden decrease in volume when subjected to wetting, referred to also as “collapse” (Wang, 2003; Xue et al., 2021). Microstructural deterioration, induced by chemicals, can cause a degradation of the macro mechanical properties (Hu et al., 2022; Wang et al., 2022b; Xue et al., 2022; Bai et al., 2019, Bai et al., 2021, Bai et al., 2021, Bai et al., 2021, Bai et al., 2022). Permeability evolution starts drawing attention in recent years as it is deemed crucial in shale gas exploration and exploitation (He et al., 2019; Nygård et al., 2006; Boulin et al., 2013; Rezaeyan et al., 2015; Wu et al., 2020; Bai et al., 2021). Wu et al. (2021) studied the permeability evolution of the original gypsum and mudstone using the triaxial compression tests and reported the main causes leading to the differences in the permeability evolution, including the failure mode and deformation character. Kou et al. (2021) investigated the failure energy and permeability evolution of fissured rock-like materials subjected to seepage pressures. The experimental results and coupling hydro-mechanical failure mechanism enhance our understanding of aspects of monitoring and controlling rock stability in the geological engineering projects in the coupling seepage-stress environments. Jiang et al. (2021) experimentally studied the evolution of pore-fracture structures and mechanism of permeability enhancement in coal under cyclic thermal shock. The findings of this study showed that cyclic thermal shock could aggravate the formation and expansion of permeability pores, connect the relatively sole fracture structures, and cause damages to the microfractures toward developing an interwoven fracture network. Li et al. (2020) analyzed the fracture permeability evolution of laminated sandstones during the rupture phase in the triaxial compression process and suggested that horizontal wells of the laminated sandstone reservoir should be set in a polyline or oblique trajectory that is likely to be intersected with the formation lamina, thereby developing an appropriate perforating direction. Lyu et al. (2021) studied the variations in pore characters of sandstone and limestone in the temperature range of 25°C-600°C based on the Katz-Thompson theory. The results showed that the permeability of limestone is nearly constant from 25°C to 400°C and is rapidly increased from 400°C to 600°C and that the permeability of sandstone can be characterized as a small fluctuation from 25°C to 400°C, followed by a gradual increase from 400°C to 570°C and subsequently a rapid increase from 570°C to 600°C.

Inorganic contaminants, including a variety of harmful metals (Otunola and Ololade, 2020; Hu et al., 2021c; Schindler et al., 2021), salts (Arocena and Rutherford, 2005; Zhang et al., 2008), acids (Wen et al., 2020; Zha et al., 2021), and alkaline substances (Seleiman and Kheir, 2018; Chen et al., 2020), and so forth are a frequent pollutant. Among the inorganic contaminants, chemical contaminant-induced accident is becoming more and more frequent and threatens seriously human health (Anae et al., 2021; Silva et al., 2021; Zhang et al., 2021). Loess soil originated in the deserts of the north (Smalley et al., 2014) and then distributed across NW China is featured with metastable microstructure (Assallay et al., 1997; Wen and Yan, 2014; Xu et al., 2018, 2021c; Shao et al., 2018; Wei et al., 2020). The cement, formed by a fibrous calcite scaffold with a clay cover, between silt particles provides a sufficient shear strength under dry conditions despite the majority of loess soil having void ratios ranging from 0.9 to 1 (Smalley and Marković, 2014). Such a problematic soil with very high void ratio is, therefore, considered susceptible to chemical contaminant permeation. A significant body of research conducted over the last 10 years has greatly enhanced our understanding of the microscopic characteristics and mechanical properties of natural soils under water and chemical actions (Gratchev and Towhata, 2011, 2013; Nguyen et al., 2013; Zhang et al., 2013, 2018, 2020; Shao et al., 2018; Lei et al., 2020; Hu et al., 2021a). The salt effect can promote the dissolution of calcite and dolomite and the cation exchange can aggravate the leaching of other cations, thereby leading to further microstructural loosening (Xu et al., 2020, 2021a, b). Given that the permeability behavior of the loess is deemed of great importance for the prevention and mitigation of chemical contamination, enhancing our understanding of the effect of seepage conditions on the microstructural evolution of loess soils is in pressing need (Chen et al., 2021; Gao et al., 2018; Hou et al., 2020; Wang et al., 2020, 2021). The authors had conducted a series of experiments that pave the way to the success of interpreting the correspondence of chemical actions with the mechanical properties of loess soils (Hu et al., 2021a). Notwithstanding that, our understanding of the linkage of water and chemical seepages with the microstructural evolution of loess soils and change in the macro permeability properties is still poor. These results not only limit the development of remediation technologies but also lead to an inability of ensuring environmental sustainability and human health safety. The objectives of this study are: (1) to conduct a comprehensive investigation into the permeability evolution of the loess under the water and sodium sulfate seepages, respectively, (2) to comment on the effect of pore-water chemistry on the microscale structure characteristics of the loess using the temporal relationship of cations concentration, X-ray diffraction and fluorescence (XRD and XRF) tests, mercury intrusion porosimetry (MIP) test, and scanning electron microscope (SEM) and (3) to reveal the mechanisms affecting the permeability evolution of the loess.

## Results

### Water seepage test

#### Permeability evolution

**Figure 1:**
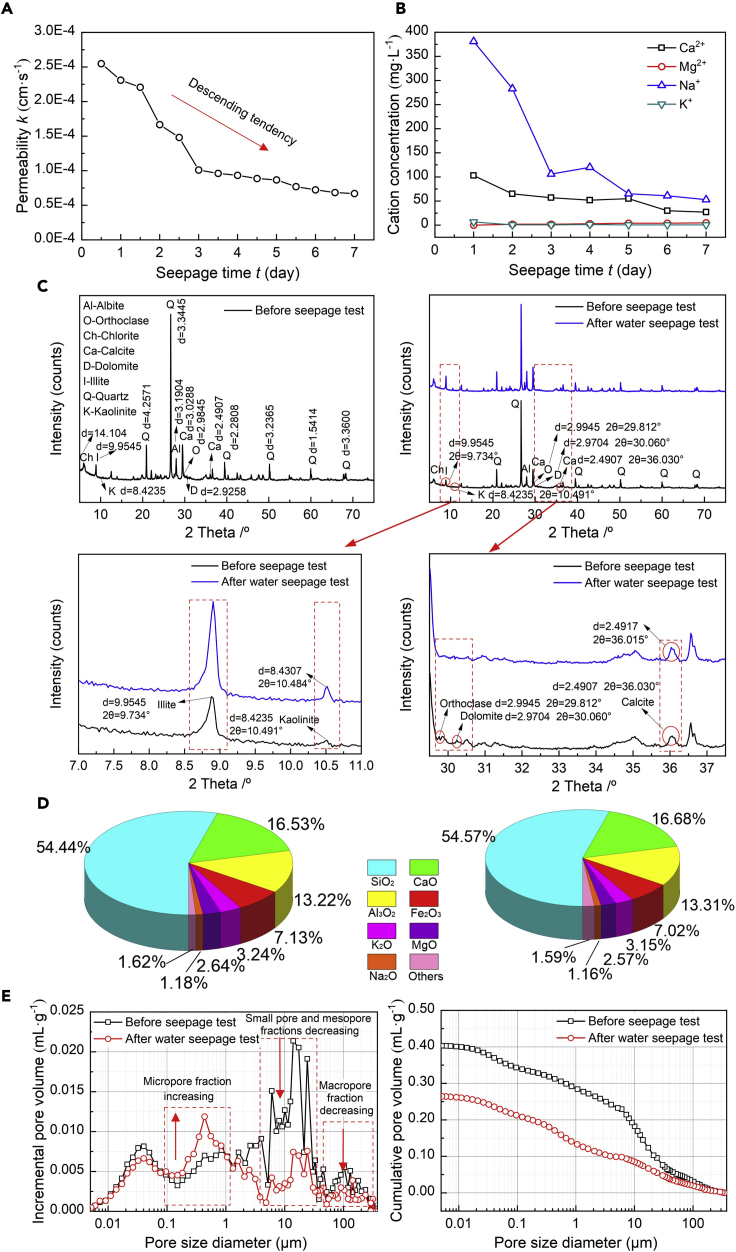
Water seepage test (A) Temporal relationship of permeability k versus seepage time t for the water seepage test, (B) temporal relationship of cation concentration versus seepage time t for the water seepage test, (C) XRD test results before and after the water seepage test, (D) XRF test results before and after the water seepage test, and (E) MIP test results before and after the water seepage test.

The test processes and physical property are shown in Figures S1 and S2. Figure 1A shows the relationship of permeability *k* versus seepage time *t* for the loess specimen subjected to the water seepage test. The value of *k* descends very quickly in the first three days of water seepage and then remains a gentle descending tendency toward the end of the water seepage test. Figure 1B shows the change in concentration for Na^+^, K^+^, Ca^2+^, and Mg^2+^ during the water seepage test. In the first three days of water seepage, the change in concentration for Na^+^ is the most significant, followed by Ca^2+^. Both Na^+^ and Ca^2+^ show a gentle descending tendency in the remaining four days. The change in concentration for Mg^2+^ is almost negligible either in the first three days or in the remaining four days. At the end of the water seepage test, the concentration of the four cations is ordered as follows: Na^+^, Ca^2+^, Mg^2+^, and K^+^.

### X-ray diffraction, X-ray fluorescence, and mercury intrusion porosimetry tests

Figure 1C shows the XRD test results for the loess specimen before and after the water seepage test. Four notable diffraction peak changes at 9.9545 Å, 2.9945 Å, 2.9704 Å, and 2.4907 Å, respectively, are detected and identified as illite, orthoclase, dolomite, and calcite. These results demonstrate that the four minerals play parts in the water seepage test and could help reveal the mechanism affecting the permeability evolution of the loess under the effect of water seepage. Figure 1D shows the XRF test results for the loess specimen before and after the water seepage test. The XRF test results supplement the XRD test results because the formation and/or disappearance of chemical compounds may trace back to the change in diffraction peak. The concentration of sodium oxide, magnesium oxide, and potassium oxide descends after the water seepage test, whereby the concentration of silicon dioxide and aluminum oxide ascends after the test. On the other hand, the incremental and cumulative pore volume curves, derived from the MIP tests, for the loess specimen before and after the water seepage test are shown in Figure 1E. The cumulative pore volume curve for the loess after the water seepage is differentiable from that for the loess before the water seepage. The most significant changes are present in a pore size range of 0.2–32 μm.

### Scanning electron microscope tests

**Figure 2:**
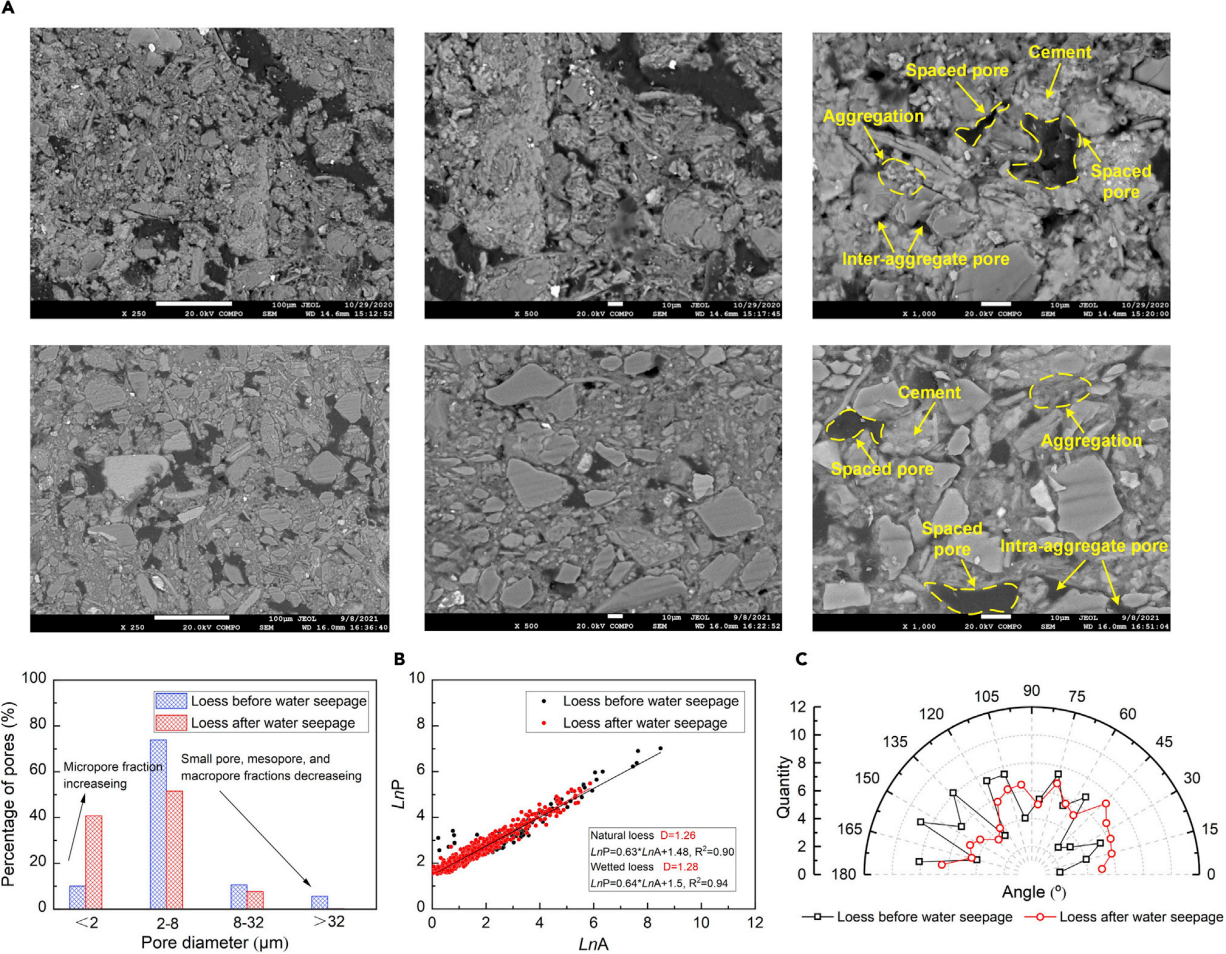
SEM test under water seepage condition (A) SEM images before (upper part) and after the water seepage test. (Lower part), (B) pore distribution and fractal dimension before and after the water seepage test, and (C) pore directionality before and after the water seepage test.

Figure 2A presents the SEM test results of the loess before and after the water seepage, and the SEM image on the left represents a magnification of 250 times, followed by a magnification of 500 times in the middle, and a magnification of 1000 times on the right. The particle morphology of the natural loess comprises granular structures. The cementation, induced by calcium carbonate and magnesium carbonate (namely “cement”) between silt particles, promotes the typical inter-particle connection forms (including point-edge and edge-edge connections) to be developed (see the upper part of Figure 2A). A microscale structure containing the afore-said characteristics is referred to also as the “metastable microscale structure,” and it is featured spaced pores, inter-particle pores, and intra-aggregate pores. The intra-aggregate pores correspond to those present in between the fine-grained particles, whereas the inter-aggregate pores refer to those separating the clay-silt aggregates. The spaced pores are present in the soil skeleton and are the largest among the three types of pores. The lower part of Figure 2A shows the particle morphology of the wetted loess. It remains nearly the same in comparison with the natural loess, but the wetting-induced collapse deformation causes the spaced pores and/or inter-particle pores to become smaller than before. The analysis of pore distribution and fractal dimension *D*, as well as pore directionality, is shown in Figures 2B and 2C, respectively. The pore distribution can be characterized as the micropore (<2 μm) fraction increasing and the small pore (2–8 μm), mesopore (8–32 μm), and macropore (>32 μm) fractions decreasing (Lei, 1987). The value of *D* owing to the effect of water seepage shows a small increase. On the other hand, the pores for the natural loess are mainly distributed in both the vertical and horizontal directions, indicating the preferential channels of pore fluid. The pores for the wetted loess appear to distribute in the horizontal direction, which dominates the flow regime of pore fluid.

### Sodium sulfate seepage test

#### Permeability evolution

**Figure 3:**
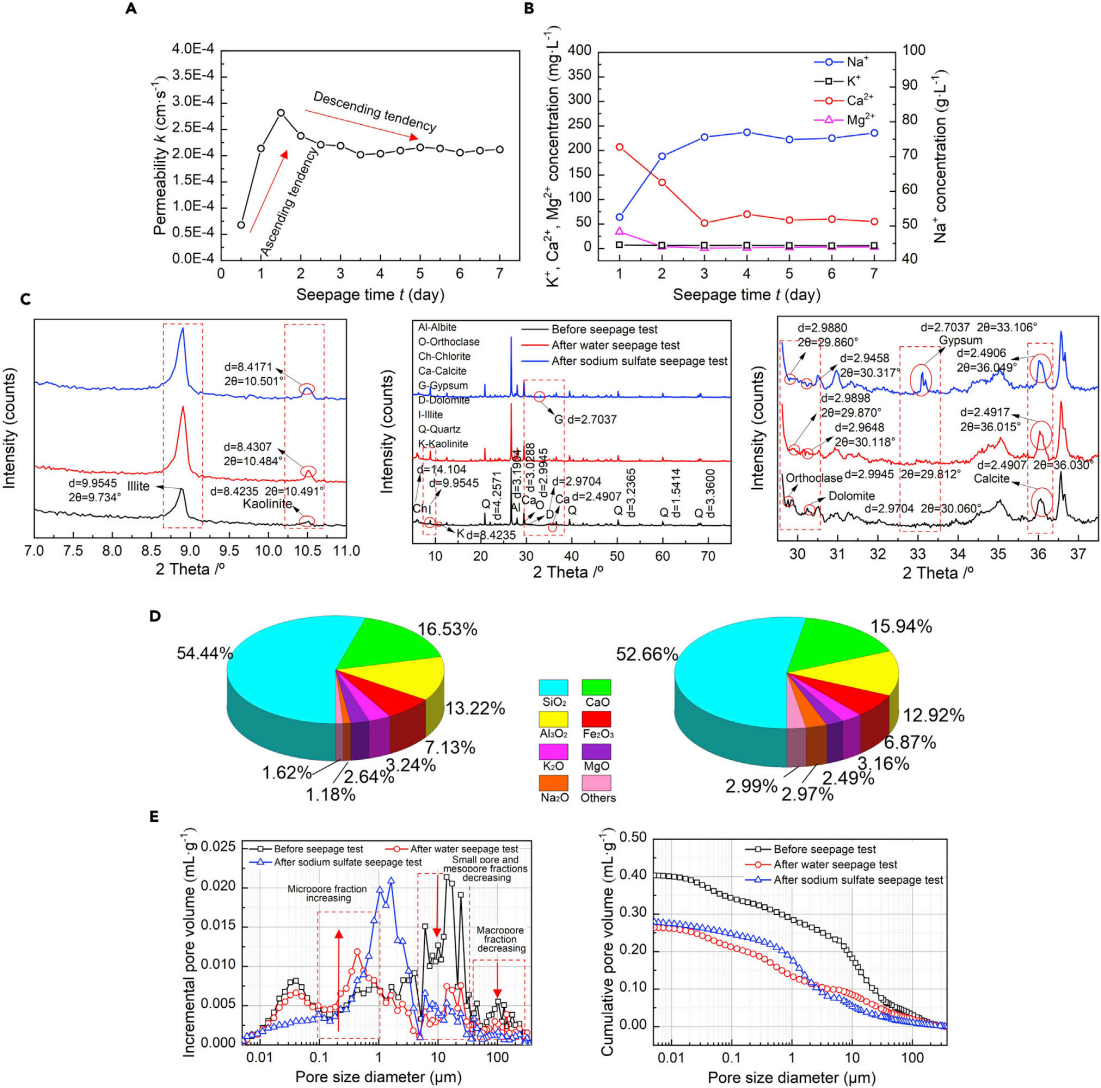
Sodium sulfate seepage test (A) Temporal relationship of permeability *k* versus seepage time *t* for the sodium sulfate seepage test, (B) temporal relationship of cation concentration versus seepage time *t* for the sodium sulfate seepage test, (C) XRD test results before and after the sodium sulfate seepage test, (D) XRF test results before and after the sodium sulfate seepage test, and (E) XRF test results before and after the sodium sulfate seepage test.

Figure 3A shows the relationship of permeability *k* versus seepage time *t* for the loess specimen subjected to the sodium sulfate seepage test. In the first one and half days of sodium sulfate seepage, the *k* value ascends rapidly. After reaching the peak, the *k* value first descends a little and remains nearly constant throughout the remaining four and half days. Figure 3B shows the relationship of cations concentration versus seepage time *t* for the loess specimen under the sodium sulfate seepage test. In contrast to the concentration of Na^+^ under the water seepage test, the concentration of Na^+^ under the sodium sulfate seepage test elevates sharply in the very beginning and remains approximately constant throughout the rest of seepage time *t*. Furthermore, the concentration of Ca^2+^ and Mg^2+^ descend in the first three days of sodium sulfate seepage and shows a little change afterward. Moreover, the concentration of K^+^ presents a negligible change throughout the whole test.

### X-ray diffraction, X-ray fluorescence, and mercury intrusion porosimetry tests

Five distinct diffraction peak changes, derived from the XRD test results, present at 9.9545 Å, 2.9945 Å, 2.9704 Å, 2.7037 Å, and 2.4907 Å, respectively, are detected and identified as illite, orthoclase, dolomite, gypsum, and calcite (see Figure 3C). The XRF test results show that the concentration of sodium oxide ascends after the sodium sulfate seepage test. Although the concentration of magnesium oxide, potassium oxide, silicon dioxide, and aluminum oxide descends after the seepage test (see Figure 3D). The cumulative pore volume curve for the loess subjected to the sodium sulfate seepage behaves not similar to that for the loess subjected to the water seepage (see Figure 3E). When under the sodium sulfate seepage, the pore sizes <8 μm show a more significant change.

### Scanning electron microscope tests

**Figure 4:**
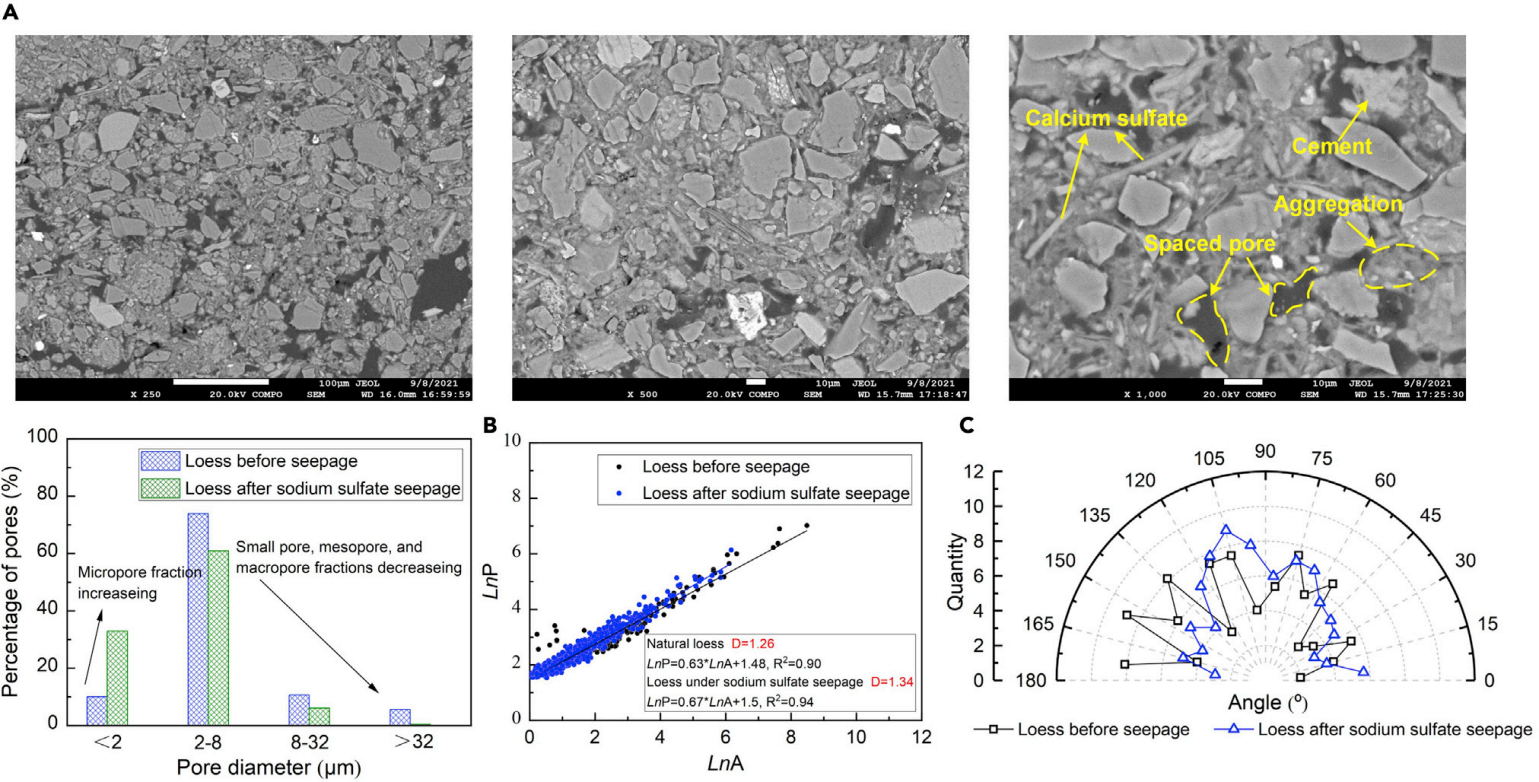
SEM test under sodium sulfate seepage condition (A) SEM images after the sodium sulfate seepage test, (B) pore distribution and fractal dimension before and after the sodium sulfate seepage test, and (C) pore directionality before and after the sodium sulfate seepage test.

Figure 4A shows the SEM test results of the loess under the effect of sodium sulfate seepage. The particle morphology does not show a substantial change in comparison with that of the wetted loess, indicating that sodium sulfate environments have a minimal impact on the particle morphology. Furthermore, the particle aggregation becomes more significant compared to that of the wetted loess, most likely because of the improvement in cementation. The edge-edge or point-edge connections, therefore, take the lead as the main inter-particle connection form. The pore distribution indicates that the micropore (<2 μm) pore fraction increases and compared to the water seepage test, the small pore (2–8 μm), mesopore (8–32 μm), and macropore (>32 μm) fractions show a relatively smaller reduction (see Figure 4B). Furthermore, the *D* value increases from 1.26 to 1.34. On the other hand, the pore directionality can be characterized as the more prominent vertical channel than the horizontal channel, indicating that the vertical channel controls the flow of pore fluid and can be considered the preferential channel (see Figure 4C).

## Discussion

### Water seepage test

Among the cations, the temporal relationship of Na^+^ concentration presents the most significant change at the beginning of the water seepage test (see Figure 1B). Albite, a rock-forming mineral, is considered the main source of Na^+^ in the water seepage test. When exposed to weak acid environments (3H_2_O+2CO_2_), it leaches sodium bicarbonate, kaolinite, and silicon dioxide (see Equation 1). Therefore, the maximum Na^+^ concentration in the temporal relationship is attributed to the leachate containing sodium bicarbonate. The descending tendency appears in the latter part of the Na^+^ concentration temporal relationship, most likely because of the ionization of sodium bicarbonate (see Equation 2). On the other hand, the temporal relationship of K^+^ concentration descends all the way toward the test end. Orthoclase discoverable in the natural loess provides the main source of K^+^. The weak acid conditions also cause orthoclase to leach, and potassium bicarbonate in the leachate promotes the formation of the maximum in the temporal relationship of K^+^ concentration (see Equation 3), and the descending tendency is formed with the ionization of potassium bicarbonate (see Equation 4).

The temporal relationship of Mg^2+^ concentration presents a slightly ascending tendency throughout the water seepage test, whereas the temporal relationship of Ca^2+^ concentration shows a descending tendency. Dolomite which is mainly consisted of Ca^2+^ and Mg^2+^ leaches when exposed to the water seepage, thereby discharging calcium bicarbonate and magnesium bicarbonate (see Equation 5). The concentration of Ca^2+^ and Mg^2+^ would have been reduced if Ca^2+^ and Mg^2+^ had been washed away (see Equations 6 and 7). Ca^2+^ or Mg^2+^ could, however, react again with dolomite, referred to also to as “dedolomitization,” discharging Mg^2+^ and precipitating calcium carbonate (see Equation 8). In other words, the dedolomitization is, in fact, a physicochemical process of transforming dolomite to calcite through the effect of Ca-rich or Mg-rich water leaching. As a result, it is considered the main cause leading to the formation of the ascending tendency of Mg^2+^ concentration and the descending tendency of Ca^2+^ concentration.

(Equation 1) $${2\text{NaAlSi}}_{3}{\text{O}}_{8}+{3\text{H}}_{2}\text{O}+{2\text{CO}}_{2}\to {\text{Al}}_{2}{\text{Si}}_{2}{\text{O}}_{5}{\left(\text{OH}\right)}_{4}+{2\text{NaHCO}}_{3}+{4\text{SiO}}_{2}$$

(Equation 2) $${\text{NaHCO}}_{3}={\text{Na}}^{+}+{{\text{HCO}}_{3}}^{-}$$

(Equation 3) $${2\text{KAlSi}}_{3}{\text{O}}_{8}+{3\text{H}}_{2}\text{O}+{2\text{CO}}_{2}\to {\text{Al}}_{2}{\text{Si}}_{2}{\text{O}}_{5}{\left(\text{OH}\right)}_{4}+{2\text{KHCO}}_{3}+{4\text{SiO}}_{2}$$

(Equation 4) $${\text{KHCO}}_{3}={\text{K}}^{+}+{{\text{HCO}}_{3}}^{-}$$

(Equation 5) $$\text{CaMg}{\left({\text{CO}}_{3}\right)}_{2}+{2\text{H}}_{2}\text{O}+{2\text{CO}}_{2}\to \text{Ca}{\left({\text{HCO}}_{3}\right)}_{2}+\text{Mg}{\left({\text{HCO}}_{3}\right)}_{2}$$

(Equation 6) $$\text{Ca}{\left({\text{HCO}}_{3}\right)}_{2}\to {\text{Ca}}^{2+}+2{{\text{HCO}}_{3}}^{-}$$

(Equation 7) $$\text{Mg}{\left({\text{HCO}}_{3}\right)}_{2}\to {\text{Mg}}^{2+}+2{{\text{HCO}}_{3}}^{-}$$

(Equation 8) $$\text{CaMg}{\left({\text{CO}}_{3}\right)}_{2}+{\text{Ca}}^{2+}\to {\text{Mg}}^{2+}+{2\text{CaCO}}_{3}$$

The formation of the kaolinite (Al_2_Si_2_O_5_(OH)_4_), induced by the leaching of orthoclase, can be characterized as its diffraction peak increasing from 8.4235 Å under no water seepage to 8.4307 Å under the condition of water seepage (see Figure 1C). Furthermore, the precipitation of calcium carbonate, induced by the dedolomitization, can also be recognized as its diffraction peak increasing from 2.4907 Å under no water seepage to 2.4917 Å under the effect of water seepage. Although the XRF test results provide supplements that are useful in exploring the mechanism affecting the permeability evolution of the loess under the effect of water seepage. The fraction of Na_2_O decreasing from 1.18% to 1.16% witnesses the ionization of sodium bicarbonate (see Figure 1D). Furthermore, the fraction of K_2_O decreases from 3.24% to 3.15%, which also provides testimony of the ionization of potassium bicarbonate. As discussed before, the dedolomitization discharges Mg^2+^ and precipitates calcium carbonate, thereby decreasing the fraction of MgO from 2.64% to 2.57% and increasing the fraction of CaO from 16.53% to 16.68%. Given that the XRF tests are on the basis of specimens retained after the seepage tests, these results are just opposite to what has already been indicated in the temporal relationships of Mg^2+^ and Ca^2+^ concentration. On the other hand, the MIP test results indicate that the pore structure for the wetted loess can be characterized as the micropore fraction increasing and the small pore, mesopore, and macropore fractions decreasing (see Figure 1E). The pore distributions, derived from the SEM images, are in line with the MIP test results (see Figure 2B). Albite leaches sodium bicarbonate, and its ionization further discharges Na^+^ and HCO_3_^−^. Na^+^ can intrude the diffuse double layer (DDL) and turn the agglomerated structure into the dispersive structure. Furthermore, the water seepage can cause the swelling and dispersion of clays. The intrusion of Na^+^ and the swelling and dispersion of clays promote the development of micropores (i.e. inter-aggregate pores) onto the cement between particles. The development of the micropores deteriorates the microstructure as well. The main cause that triggers the decrease in small pore, mesopore, and macropore fractions following the test may be ascribed to the dedolomitization. Given that the dedolomitization consumes Ca^2+^ and discharges Mg^2+^ as well as precipitated calcium carbonate, calcite is expected to precipitate onto the small pore, mesopore, and macropores toward decreasing the small pore, mesopore, and macropore fractions. The micropore fraction increasing and the small pore, mesopore, and macropore fractions decreasing increase the complexity of the microstructure (see Figure 2B). The pores present in the natural loess do not show a distinct orientation. Given the transformation of the inter-aggregate pores into the intra-aggregate pores when subjected to the water seepage test, the pore directionality appears to behave as similar as before (see Figure 2C). Such transformation eases the effect of inundation on the pore directionality. These results lead to a conclusion that the permeability of the loess subjected to the effect of water seepage descends all the way toward the test end (see Figure 1A).

On the whole, the Na_2_O and K_2_O fraction decreasing witnesses the ionization of sodium bicarbonate and potassium bicarbonate. When exposed to weak acid environments, sodium bicarbonate discharges Na^+^, and the intrusion of Na^+^ into the double layer turns the agglomerated structure into the dispersive structure. Although the intrusion of Na^+^ and the swelling and dispersion of clays promote the formation of micropores onto the cement, the dedolomitization, however, causes calcium carbonate to precipitate onto the small pores, mesopores, and macropores. The descending tendency of Ca^2+^ concentration and the ascending tendency of Mg^2+^ concentration provide testimony of the dedolomitization. It can, therefore, be summarized that the precipitation of calcite by the dedolomitization plays a leading role in modifying the micro pore fraction and degrading the macro permeability of the loess under the water seepage.

### Sodium sulfate seepage test

The temporal relationship of Na^+^ concentration shows the most notable change at the early stage of the sodium sulfate seepage test. Unlike the water seepage test, the sodium sulfate seepage causes the temporal relationship of Na^+^ concentration to behave in an ascending tendency (see Figures 1 and 3B). This is because the involved chemical reactions, including Equations 1 and 2, reverse at high Na^+^ concentrations. Furthermore, the diffraction peak decreasing from 8.4307 Å under the water seepage to 8.4171 Å under the sodium sulfate seepage indicates the consumption of kaolinite, induced by the reversed chemical reactions (see Figure 3C). The fraction of SiO_2_ decreasing from 54.44% to 52.66% and the fraction of Al_2_O_3_ decreasing from 13.22% to 12.92% provide testimony of the consumption of kaolinite (see Figure 3D).

The temporal relationship of K^+^ concentration under the sodium sulfate seepage is rather similar to that under the water seepage (see Figures 1 and 3B). Furthermore, the sodium sulfate seepage appears to aggravate the leaching of orthoclase as the diffraction peak is reduced from 2.9898 Å under the water seepage to 2.9880 Å under the sodium sulfate seepage (see Figure 3C). The leaching of orthoclase by the sodium sulfate seepage can also be characterized as the fraction of K_2_O decreasing from 3.24% to 3.16% (see Figure 3D). On the other hand, given the dolomitization (see Equation 9), the temporal relationship of Ca^2+^ and Mg^2+^ shows a descending tendency (see Figure 3B). In addition to Ca^2+^ and Mg^2+^, sulfate ions also take part in the dolomitization process and are believed to aggravate the leaching of dolomite. The diffraction peak decreasing from 2.9648 Å under the water seepage to 2.9458 Å under the sodium sulfate seepage gives testimony to the enhancement of the leaching of dolomite (see Figure 3C). Furthermore, the diffraction peak decreasing from 2.4917 Å under the water seepage to 2.4906 Å under the sodium sulfate seepage witnesses the reduction in the precipitation of calcium carbonate. The reduced precipitation of calcium carbonate can also be verified through the fraction of CaO decreasing from 16.53% to 15.94% (see Figure 3D). Despite that, the sulfate ions could also react with Ca^2+^ in the dolomitization process toward discharging calcium sulfate (i.e. gypsum) (see Equation 10). The XRD test identifies the gypsum at 2.7037 Å. The small pore, mesopore, and macropore fractions decrease witness the precipitation of gypsum despite the dissolution of calcite by the dolomitization (see Figure 4B). Given that sodium sulfate can discharge Na^+^ by its dissolution (Equation 11) and Na^+^ will invade the DDL, the salt-induced swelling phenomenon is considered as the main cause leading to the micropore fraction increasing. The pores do not show a distinct orientation, although it becomes narrower compared to that of the natural loess (see Figure 4C). This is attributed to the fissures that propagate along a direction parallel to the overburden pressure under the salt-induced swelling phenomenon. The dissolution of calcite by the dolomitization not only modifies the micro pore fraction but also increases the macro permeability (see Figure 3A).

(Equation 9) $${2\text{CaCO}}_{3}+{\text{Mg}}^{2+}\to \text{CaMg}{\left({\text{CO}}_{3}\right)}_{2}+{\text{Ca}}^{2+}$$

(Equation 10) $${\text{Ca}}^{2+}+{{\text{SO}}_{4}}^{2-}\to {\text{CaSO}}_{4}$$

(Equation 11) $${\text{Na}}_{2}{\text{SO}}_{4}={2\text{Na}}^{+}+{{\text{SO}}_{4}}^{2-}$$

In short, the decrease of SiO_2_ and Al_2_O_3_ fraction provide testimony to the consumption of kaolinite. The temporal relationships of K^+^ concentrations indicate the consumption of albite, whereas for Mg^2+^ their temporal relationship indicates the leaching of dolomite by the dolomitization. Considering Ca^2+^ discharged can react again with sulfate ions, their descending tendency is also attributed to the dolomitization. The small pore, mesopore, and macropore fractions decreasing witness the precipitation of calcium sulfate (gypsum), whereas the micropore fraction increasing indicates the dissolution of calcite. As a result, the dissolution of calcite by the dolomitization modifies the micro pore fraction toward increasing the macro permeability of the loess under the sodium sulfate seepage (Xu et al., 2020, 2021a, 2021b).

Inappropriate disposal of waste resulting from mining activities is thought as essential for promoting the effect of pore-water chemistry (i.e. dolomitization), causing a modification of the microscale pore fraction and elevating the potential of land degradation. Land degradation refers to a loss of the productive capacity of soils and is considered to be a global challenge that influences especially rural communities and smallholder farmers through food insecurity, environmental hazards, and the loss of biodiversity and ecosystem services. As land is degraded, soil carbon and nitrous oxide are discharged into the atmosphere. This makes land degradation one of the most important contributors to climate change. Land degradation is happening at a remarkably fast pace, contributing to a dramatic decline in the productivity of croplands and rangelands around the world. The problem is particularly severe in the semi-arid and arid regions. Women and children who rely on drylands for their livelihoods are the most vulnerable to the impacts of land degradation. As a result, managing land more sustainably to reduce degradation and increasing rates of land restoration are considered of great necessity. The two ends converge to give an aggressive control of land degradation. According to the finding of this work, biomineralization technology may be used to precipitate calcium carbonate through catalyzing urea hydrolysis using the ureolytic bacteria (Hu et al., 2021b; Xue et al., 2022; Wang et al., 2022a, c). The precipitation of calcium carbonate not only impedes the dissolution of calcite by the dolomitization but also reduces rates of land degradation. Furthermore, biomineralization technology is also deemed effective in solidifying heavy metals where a multilayer structure of calcium carbonate binds heavy metals to precipitate onto the anionic surfaces of the cell wall. To conclude, biomineralization technology and other sustainable land management practices provide an opportunity to sustain and rebuild productive areas, increase the prospects for food security for smallholders and rural communities, and mitigate the impacts of land degradation.

### Conclusions

The permeability evolution of the loess under the water and sodium sulfate seepages are studied, respectively. The microscale pore fraction modifications are in close relation with the macroscale permeability evolution. Based on the results and discussion, some main conclusions can be drawn as follows:

(1) The descending tendency of Ca^2+^ concentration and the ascending tendency of Mg^2+^ concentration is attributed to the dedolomitization. Although the intrusion of Na^+^ into the DDL and swelling and dispersion of clays turn the agglomerated structure into the dispersive structure and form the micropores onto the cement, the precipitation of calcite by the dedolomitization reduces the small pore, mesopore, and macropore fractions. The precipitation of calcite modifies the micro pore fraction and degrades the macro permeability of the loess under the water seepage.
(2) Ca^2+^ and Mg^2+^ concentrations increasing are mainly owing to the dolomitization. Ca^2+^ concentration owing to the leaching of calcite would have been increased if gypsum had not been produced. Although the formation of gypsum reduces the small pore, mesopore, and macropore fractions, the hydrolysis of sodium sulfate increases the DDL thickness, referred to also as the “salt-induced swelling” phenomenon, thereby producing micropores. Also, the dissolution of calcite by the dolomitization modifies the micro pore fraction toward enhancing the macroscale permeability of the loess under the sodium sulfate seepage.
(3) The XRD and XRF tests provide testimony of the physicochemical reactions and are useful in exploring the mechanisms affecting the permeability evolution of the loess under the water and sodium sulfate seepages. While the MIP and SEM tests mainly aim to stand out the modifications of pore fraction which determine the macroscale permeability evolution. Although the present work is based upon indoor experiments, its findings highlight the effect of water and sodium sulfate seepage conditions on the microstructural evolution of loess, providing critical insights into land degradation prevention and control in semi-arid and arid regions around the world.

### Limitations of the study

In this study, the authors conducted a comprehensive investigation into the permeability evolution of the loess under the water and sodium sulfate seepages, respectively, commented on the effect of pore-water chemistry on the microscale structure characteristics of the loess using the temporal relationship of cations concentration, XRD and XRF tests, MIP test, and SEM, and revealed the mechanisms affecting the permeability evolution of the loess. However, the permeability evolutions and the microscale structure characteristics of the loess under acid and alkaline environments have not investigated yet. Further works in relation to the said inadequacies are ongoing and the results would be presented in another paper.

## STAR★Methods

### Key resources table

**Table:** 

REAGENT or RESOURCE	SOURCE	IDENTIFIER
**Chemicals, peptides, and recombinant proteins**		

Sodium Sulfate (Analytical grade)	Greagent	CAS:7757-82-6

**Software and algorithms**		

Particles (Pores) and Cracks Analysis System (PCAS 2321)	Nanjing University	http://acei.cn/program/PCAS

**Other**		

Bruker AXS X-ray diffractometer (D8 Advance)	Bruker	https://www.directindustry.com/prod/bruker-axs-gmbh/product-30028-1821023.html
X-ray fluorescence (XRF) spectrometer (RIGAKU ZSX Priums)	PANalytical	https://www.malvernpanalytical.com.cn/products/category/x-ray-fluorescence-spectrometers/
Scanning electron microscopic JSM-7610F	JEOL	https://www.antpedia.com/ibook126/p/35497-p.html
Mercury intrusion porosimetry AutoPore IV 9500	U.S. Micro	https://www.instrument.com.cn/netshow/C222916.htm
K^+^, Ca^2+^, Mg^2+^ concentration measurement (HANNA HI96752)	Italy HANNA	https://b2b.baidu.com/land?id=c7f27003d4caf795e8e414ac8244cd0c10
Na^+^ concentration measurement (HANNA HI931101)	Italy HANNA	https://b2b.baidu.com/land?id=0afde10d82ff69b9bfb20c280239d28810
GDS triaxial permeameter	U.K. GDS	http://sgch.xauat.edu.cn/dxyqweb/yingxin/yemian/sbzs.jsp

### Resource availability

#### Lead contact

Further information and requests for resources should be directed to and fulfilled by the lead contact, Wen-Chieh Cheng (w-c.cheng@xauat.edu.cn).

#### Materials availability

This study did not generate new unique reagents.

### Method details

#### Materials

Located in the southernmost Chinese Loess Plateau and in the northern foothills of the Qinling Mountain, Lantian County, Shaanxi Province is famous for its intensive mining activities (see Figure S1A). The intensive mining activities also cause a series of outcrops available in Lantian County, Shaanxi Province. On the other hand, East Asian Monsoon and Siberian High control local climate, and eluviation and reprecipitation become more intensive, which forms distinct loess/paleosol profiles (see Figure S1A). These results lead us to choose Lantian County as our sampling spot to investigate the permeability evolution of loess under the effect of water and inorganic contaminant. The Q_3_ loess block samples of 30 cm in length, 30 cm in width, and 20 cm in height from depths of 4.5 m below the surface were retrieved where a distinct loess/paleosol profile appears (see Figures S1A and S1B). In light of this, the sampling won’t disturb by the paleosol, and its accuracy was largely improved. On the other hand, the sampling depth of 4.5 m aimed to prevent possible interference, induced by cultivation and post-exposure alteration, by removing the surface portion of the said profile. The sampling depth is in line with that adopted by Xue et al., (2011). They were wrapped with multiple layers of preservative films before transporting back to the laboratory for testing. Then the samples were sealed with hot wax and placed into a chamber. The physical properties, including unit weight (γ), water content (ω_n_), specific gravity (G_s_), liquid limit (ω_L_), and plastic index (PI), for the loess were evaluated in accordance with ASTM D2216, D854, and D4318 respectively (ASTM D2216-19, 2019; ASTM D854-14, 2014; ASTM D4318-17, 2017). According to ASTM D6913 (ASTM D6913-04, 2009), the particle size distribution curve for the loess indicates that sand, silt, and clay have fractions of 3.3%, 87.4%, and 9.3% respectively (see Figure S1A). While ω_L_ and PI for the loess are 31.6 and 12.1% respectively (see Figure S2B). The tested loess is classed as the low plasticity clay (CL), in accordance with the unified soil classification system (USCS). The physical properties of the loess are tabulated in Table S1, and the chemical and mineral compositions of the loess are shown in Table S2.

#### Water and sodium sulfate seepage tests

Prior to the water and sodium sulfate seepage tests, deionised water and sodium sulfate solution with a concentration of 2 mol/L (National Bureau of Statistics, 2001; National Bureau of Statistics, 2018) were prepared. The water and sodium sulfate seepage tests were conducted using the GDS permeameter (GDS-PERM) (see Figure S1C). The cylinder specimens were first back pressure saturated toward determining the Skempton’s pore pressure parameter B. The value of B is set at 0.98. Then the cylinder specimens were isotropically consolidated at 200 kPa. The measured volumetric change of specimens approaching zero or negligible indicated a satisfactory degree of consolidation. The top and bottom back pressures were imposed incrementally not only to prevent restructuring of specimens but to provide a pressure gradient of 10 kPa to seepage tests. The concentration of four cations, including Na^+^, K^+^, Ca^2+^, and Mg^2+^, while performing the water and sodium sulfate seepage tests, was measured every 24 h. While measurements of the electric conductivity (EC) and pH were taken using the Hanna Instruments EC and pH probes respectively. Assuming the water and sodium sulfate seepages are in line with the Darcy’s law, the permeability *k* for the cylinder specimens can be backcalculated.

#### Micro XRD, XRF, SEM, and MIP tests

Minerals primarily included in the loess were analyzed by Bruker AXS X-ray diffractometer (D8 Advance) (see Figure S1D). The change in the fraction of minerals can be evaluated by comparing the X-ray diffraction (XRD) test results before the tests to those after the tests. The scanning rate and stride width being 2°/min and 0.02° respectively were applied to the XRD tests. On the other hand, chemical compounds that originate the loess or are formed under the effect of pore-water chemistry were analyzed using PANalytical Axios X-ray fluorescence (XRF) spectrometer (RIGAKU ZSX Priums), thereby highlighting their impacts on the permeability evolution. The microscale structure characters of the specimens under the water and sodium sulfate seepages were investigated through scanning electron microscope (SEM) tests respectively (see Figure S1E). For this reason, films were prepared and applied to the SEM tests. The cubic specimens were dried at 60° and strengthened with a solidification agent, trimming them to a film dimension of 2 mm high by 5 mm wide by 5 mm long. The films were then ground with sandpaper of different finenesses and polished by alumina solution, highlighting the microstructural characteristics. Fine grinding was conducted following rough grinding. Finally, the films were coated with a gold foil for the sake of film installation. The SEM images were taken using JEOL scanning electron microscopic JSM-7610F and processed using Particles (Pores) and Cracks Analysis (PCAS) System. The PCAS software makes objects counting, classification, and measurement easier toward characterising the pore structure morphology, inter-particle connection, pore distribution and directionality, and structural complexity (Xu et al., 2021c). Mercury intrusion porosimetry (MIP) tests applied to analyze pore distribution were performed toward comparing the pore size diameter before and after seepage (see Figure S1F). The surface tension of mercury is 4.85 × 10^−1^ Nm^−1^ at 20 degree celsius, while the contact angles and equilibrium time for each intrusion or extrusion are 130° and 5 s respectively. The microscale test results are considered beneficial in improving our understanding of the permeability evolution of the loess under the effect of water and sodium sulfate seepages.

## Data Availability

• All data reported in this paper will be shared by the lead contact upon request. • This paper does not report original code. • Any additional information required to reanalyze the data reported in this paper is available from the lead contact upon request.
