# Supplementary material for: Effect of seepage conditions on the microstructural evolution of loess across north-west China
Source: iScience. 2022 Jun 30;25(8):104691. doi: 10.1016/j.isci.2022.104691 (PMC9287809; doi:10.1016/j.isci.2022.104691)
Supplement: Document S1. Figures S1 and S2 and Tables S1 and S2 [file mmc1.pdf]

iScience, Volume *25*

Supplemental information

Effect of seepage conditions on the microstructural evolution of loess across north-west China

Lin Wang, Wen-Chieh Cheng, Wenle Hu, Shaojie Wen, and Sen Shang

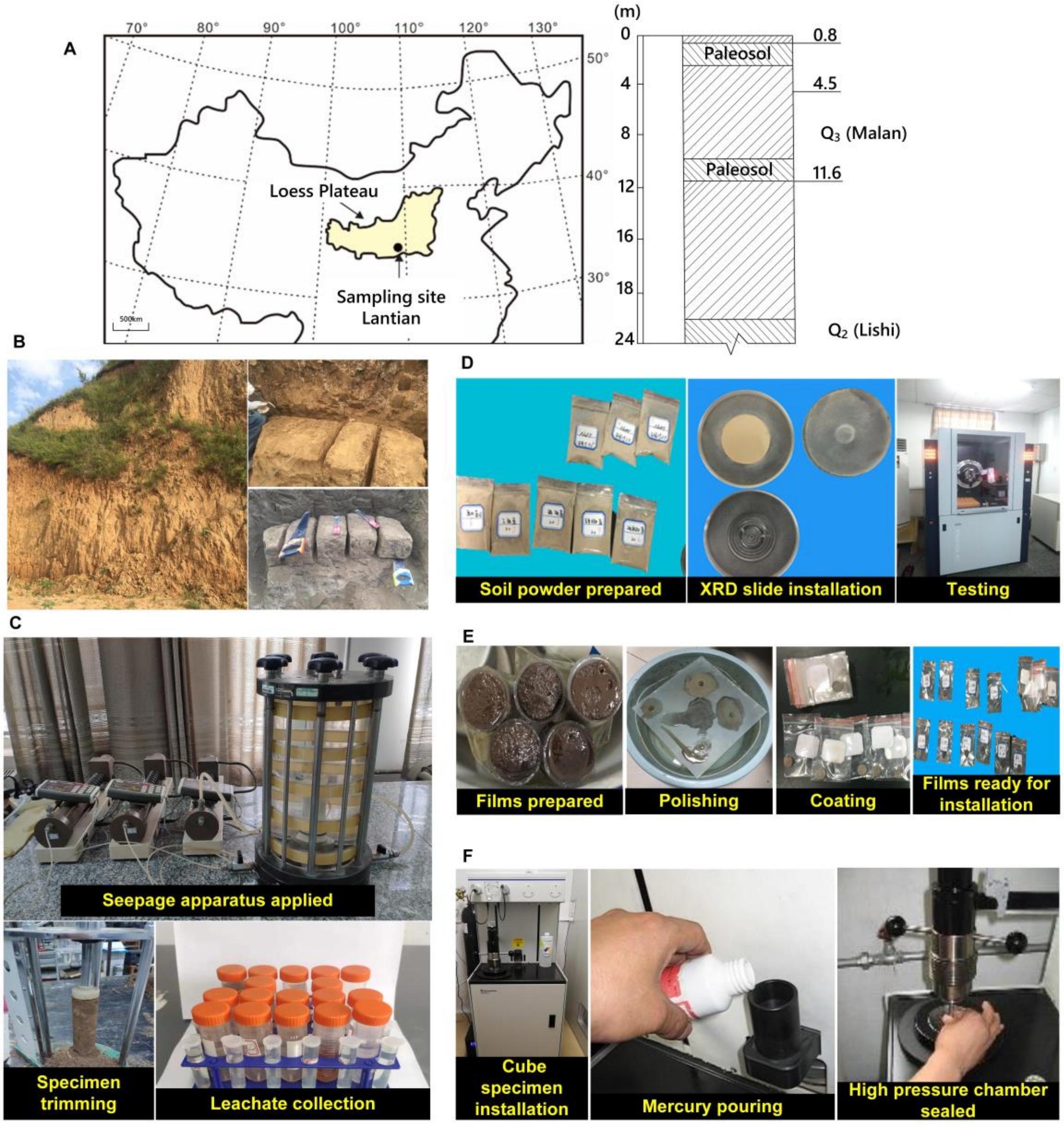

Figure S1. Sample location and test processes, Related to Figures 2 and 4. (A) Location map and geological profile, (B) sample collection, (C) seepage test and leachate collection, (D) XRD test, (E) SEM test, and (F) MIP test

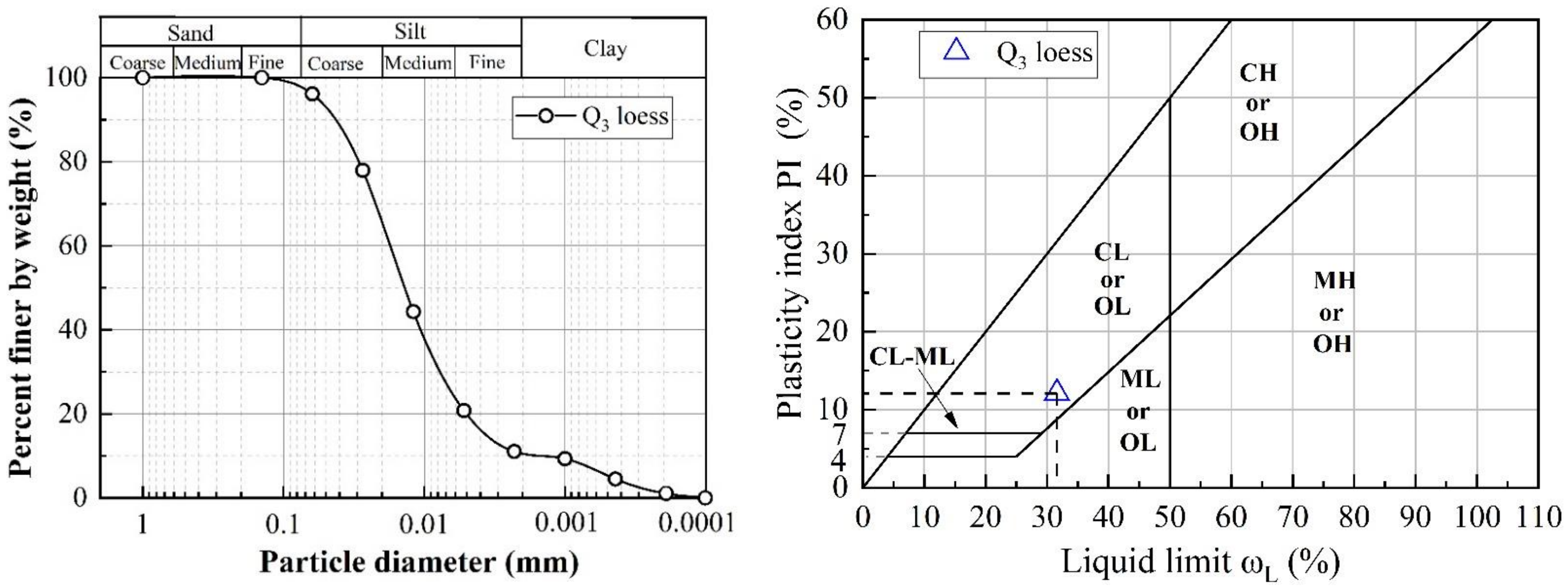

A B

Figure S2. Soil classification, Related to Figures 1 and 3. (A) Particle size distribution curve and (B) liquid limit and plasticity index

Supplemental Tables

Table S1 Physical properties of Q_3 loess. Related to STAR methods.

Physical parameter	Value
Sand (%)	3.3
Silt (%)	87.4
Clay (%)	9.3
Void ratio, e	0.898
Unit weight, γ (kN/m^3)	16.2
Specific gravity, G_s	2.69
Water content, ω_n (%)	16.5
Liquid limit, ω_L (%)	31.6
Plasticity index, PI (%)	12.1
USCS symbol	CL

Note: USCS is an abbreviation of the Unified Soil Classification System.

Table S2. Chemical and mineral compositions of Q_3 loess. Related to STAR methods.

Compound	Value (%)	Element	Value (%)
SiO_2	54.44	O	44.60
CaO	16.53	Si	25.45
Al_2O_3	13.22	Ca	11.82
Fe_2O_3	7.13	Al	7.00
K_2O	3.24	Fe	4.99
MgO	2.64	K	2.69
Na_2O	1.18	Mg	1.59
Others	1.62	Na	0.87
